# Thiol–acrylate side-chain liquid crystal elastomers

**DOI:** 10.1039/d2sm00547f

**Published:** 2022-06-03

**Authors:** Hongye Guo, Mohand O. Saed, Eugene M. Terentjev

**Affiliations:** Cavendish Laboratory, University of Cambridge J. J. Thomson Avenue Cambridge CB3 0HE UK emt1000@cam.ac.uk

## Abstract

The Michael addition ‘click’ chemistry was used to graft acrylate-terminated mesogenic groups onto the polysiloxane backbone polymer chain with thiol functional groups, with a constant 15% fraction of diacrylate reacting monomers as crosslinkers. Three different types of mesogens were used, and also their 50 : 50 mixtures, and in all cases we have obtained the smectic-A phase of the resulting liquid crystalline elastomer. Using X-ray diffraction, calorimetry and dynamic mechanical analysis, we investigated the relationship between the molecular structure of mesogenic side groups and the structure and properties of the elastomers. The shape-memory of smectic elastomers was verified. The unusual features were the semi-crystalline nature of elastomers with non-polar mesogens and the clear role of side-by-side rod dimerization of polar mesogens leading to a higher smectic layer spacing. We investigated the evolution of the smectic alignment on uniaxial stretching along the layer normal and identified two distinct ways in which the elastomer responds: the coarsened Helfrich–Hurault zig-zag layer texture and the large-scale stripe domains of uniform layer rotation in the systems with lower order parameter and the associated layer constraints.

## Abstract 2

The Michael addition ‘click’ chemistry was used to graft acrylate-terminated mesogenic groups onto the polysiloxane backbone polymer chain with thiol functional groups, with a constant 15% fraction of diacrylate reacting monomers as crosslinkers.

## Graphical abstract

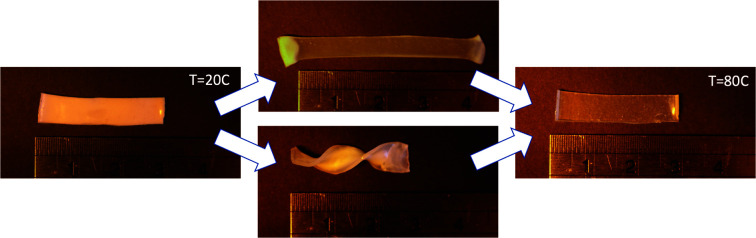

## 1 Introduction

Liquid crystalline elastomers (LCEs) have been at the forefront of scientific research for over 40 years, ever since Finkelmann synthesised the first side-chain LCE based on polysiloxanes.^1^ In the following decades, many other types of both main chain and side chain LCE networks have been explored. Owing to the exceptional mechanical characteristics of LCEs, such as thermal actuation^2–4^ and “soft elasticity”,^5,6^ this field has become a focus of many disciplines, from physicists analyzing complex interaction between orientational order and rubber elasticity to chemists developing new materials, and to engineers looking for practical applications such as artificial muscles,^7^ smart textiles,^8^ sensors^9^ and soft robotics.^10^ There are two main classes of LCEs, with most of the work focussing on their nematic phase. The smectic phase of LCEs has traditionally also been interesting and important, both from an experimental standpoint, offering several unique properties and characteristics,^11–14^ and from a theoretical angle, due to their distinct coupling between network crosslinks and the smectic layers.^15–17^

Most of the early LCEs have been of the side-chain topology, following the original ideas of Finkelmann, where the mesogenic units were joined with the siloxane backbone through the hydrosilylation reaction. This chemistry was challenging, as it required a very precise temperature and humidity controls, and was prone to (platinum) catalyst poisoning. However, after the breakthrough in LCE chemistry in 2015^18^ when the robust ‘click’ chemistry of thiol–enes and thiol–acrylates was introduced into this field, the majority of LCEs produced has become main-chain, mainly in the nematic phase. Many important advances were made with these new LCE materials;^19,20^ however, the siloxane groups have become much less frequent in the new generation of LCEs. Yet, the use of siloxane is attractive, because, since the pioneering work of Finkelmann, the siloxane backbone in side-chain LCEs was the cause for low glass transition and the excellent mechanical properties of the materials.^21^ Also, it was recently found that the siloxane segments allow for the dynamic bond exchange,^22^ thus offering an opportunity for plastic re-moulding of siloxane LCEs, similar to vitrimers.^23^

Note that due to the chemical incompatibility of siloxane and carbon-based moieties, the side-chain siloxane LCEs have a specific propensity to form the smectic-A phase. Many of the original side-chain siloxane LCEs have been nematic, but that was achieved by having a short, and often bent spacer between the mesogenic rod-like unit and the flexible backbone, as well as the side-on mesogen topology.^24^

**Fig. 1:**
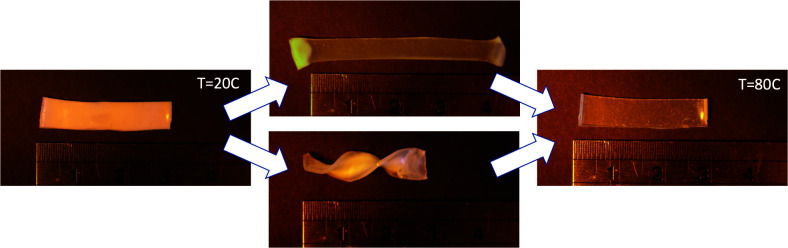
The shape memory effect in side chain LCEs in action. Left: A polydomain sample at 20 °C. Middle: The same sample aligned horizontally (top) or formed into a helix. Right: The original shape was recovered by heating to 80 °C.

Here we bring together the modern click chemistry of thiol–acrylate Michael addition and the use of polysiloxane polymer backbone to synthesise a range of side chain LCEs by attaching mesogenic acrylates to thiol-containing polysiloxane chains. These LCEs exhibit the classical side chain LCE shape memory effect as shown in Fig. 1.^11^ One of the big advances in the design of LCE materials has been the ability to use commercially available off-the-shelf components and ingredients, thus allowing even non-specialist research groups to produce their own elastomers. It has been helped by the increasing availability of reacting mesogenic monomers on the suppliers market. Driven by this motivation, here we have worked with exclusively commercial components, with all our chemistry confined to a one-pot ‘click’ reaction that is robust enough and fast enough for highly reproducible outputs.

## 2 Experimental section

### 2.1 Materials

Three acrylate-terminated mesogenic reacting monomers and a di-acrylate reacting crosslinker were used in this work.

4-(6-Acryloyloxyhexyloxy)-benzoic acid (4-cyanophenyl ester) (designated RM105 by the supplier) was purchased from Daken Chemical Co.

4-Methoxyphenyl 4-((6-(acryloyloxy)hexyl)oxy)benzoate (designated RM23 by the supplier) was purchased from Wilshire Technologies Inc. It differs from RM105 by the highly polar –CN group at the end of the mesogen.

3-[(3′-Cyanobiphenyl-3-yl)oxy]propylacrylate, designated CB3A to reflect the highly polar cyanobiphenyl mesogen, with a shorter spacer was purchased from Daken Chemical Co.

Finally, the di-acrylate mesogenic crosslinker RM82 was purchased from Daken Chemical Co. RM82 is a very commonly used reacting mesogen, which could be sourced from many suppliers.

**Fig. 2:**
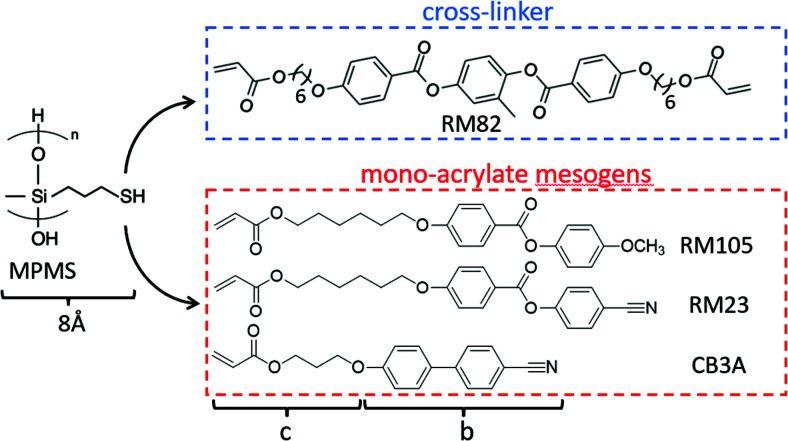
Chemical structures of side chain LCE constituents (*b* and *c* are defined as the physical length of the rigid “rod” and the flexible “tails”, respectively). The polysiloxane backbone polymer chain with thiol functional groups (MPMS), with the average chain length *n*= 80 (molecular weight 4000–7000 g mol^−1^); side-chain acrylate reacting mesogens RM105 (*b* = 13.7 Å, *c* = 12.1 Å), RM23 (*b* = 13.0 Å, *c* = 12.1 Å) and CB3A (*b* = 11.1 Å, *c* = 9.1 Å); the mesogenic crosslinker is di-acrylate RM82. The thiol functional groups on MPMS react with acrylate groups on both the mono-acrylate mesogens and the crosslinker RM82 to form a complete network.

The siloxane polymer backbone with thiol-terminated reacting side groups, (mercaptopropyl)methylsiloxane (MPMS), was purchased from Gelest, Inc. Fig. 2 shows the chemical structures of the reacting materials used in this work. Toluene was purchased from Sigma-Aldrich. Dipropylamine (DPA) was purchased from Sigma-Aldrich and used as the catalyst for the thiol–acrylate Michael addition reaction. Note that in the original Finkelmann's side-chain polysiloxane LCEs,^2,25,26^ as well as Keller's,^24^ we mostly used vinyl-terminated mesogens, and it was clearly established that the longer the spacers, the stronger the smectic propensity is in the material.

### 2.2 Synthesis procedure

For this work, a “one pot reaction” route was used to produce polydomain LCE plates in a Teflon mould, with three mesogenic acrylates CB3A, RM23, and RM105, and mixed combinations of them (equal molar amount mixing between each pair). A constant crosslinking density of 15 mol% RM82 was selected to keep this variable fixed.

First, the selected mesogen species and the crosslinker RM82 were dissolved in toluene (40 wt% of mesogen species), assisted by gentle heating and vigorous mixing. Then, the network backbone MPMS (4 mmol) was added to the solution and mixed until a homogeneous solution was achieved. The catalyst DPA (0.5 wt%) was first diluted in toluene (20 wt% of mesogen species) and then quickly dispersed into the solution using a Vortex mixer. Air bubbles were removed under vacuum. The solution was injected into a mould (sealed with tape to prevent solvent evaporation) and allowed to cure in an oven at 30 °C for 12 hours (or overnight) *via* the thiol–acrylate Michael addition reaction.

**Fig. 3:**
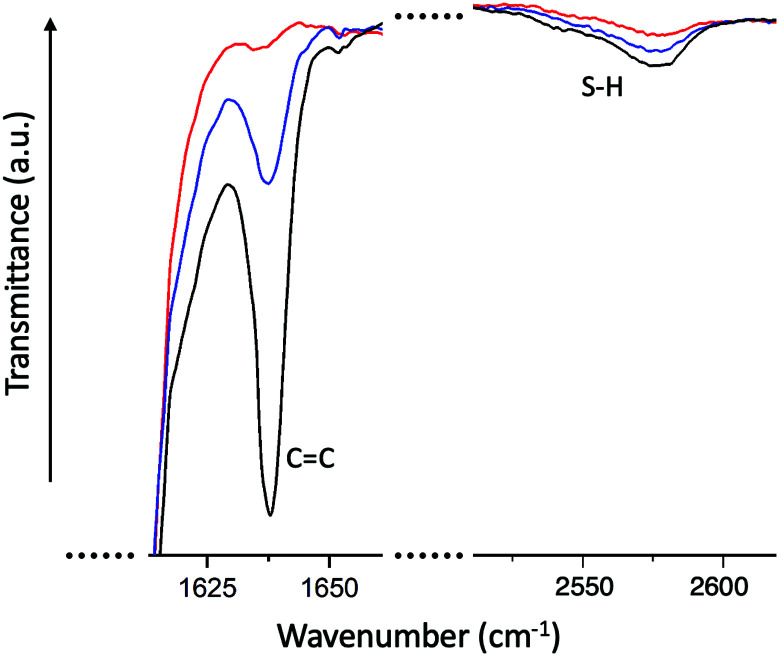
FTIR signatures of transmission peaks of –SH and of –C=C bonds in the reacting mixture (in this case, for the RM105 elastomer reaction). The curves are for *t* = 0 (for which, as a model, we used the reacting mixture without catalyst), for 5 minutes, and for 3 hours of reaction. The acrylate peak disappears completely in 3 hours, confirming the completion of the reaction. The thiol peak does not disappear completely, which is a known feature.^27,28^

To verify the completion of the reaction, we have monitored the infrared spectra (FTIR) of the reacting mixture (between glass slides) over time. Fig. 3 illustrates how the transmittance peaks corresponding to the –SH and the –C=C bonds are diminishing over the reaction time. We only show a coarse time point to confirm that the thiol–acrylate Michael addition reaction is complete in 3 hours. The FTIR signature of the thiol–acrylate Michael addition has been extensively examined in the literature, with the consistent finding that the acrylate resonance peak tends to disappear on completion, while the thiol resonance (at 2575 cm^−1^) tends to retain a residual peak. It is tempting to consider a possibility of some acrylate homopolymerization, leaving some thiols unreacted; however, the temperature is not enough for the acrylates to react, and NMR studies have shown that this hypothetical imbalanced acrylate and thiol conversion is not taking place under the reaction conditions.^27^ And, of course, the ‘click’ chemistry is not allowing such stray reaction routes to any significant extent. The observation of a residual thiol peak for negligible thiol concentrations is accepted as a feature at this resonant frequency.

### 2.3 Physical characterization

Differential scanning calorimetry (DSC) was performed on all samples to study phase transitions; we used a DSC4000 instrument from PerkinElmer. Samples were first heated to 100 °C to erase the effects of thermal history, followed by three cycles of cooling/heating between −50 and 100 °C, at a rate of 10 °C min^−1^ with a soak time of 2 minutes at each terminal temperature. The thermodynamic equilibrium *T*_c_ was obtained at the onset of phase transition.

Wide- and small-angle X-ray analyses were performed on all samples at room temperature using a Philips PW-1830 diffractometer with the main CuK_α_ wavelength of 0.154 nm. The data were collected using a wide-area detector (using a 16.8 MP sCMOS GSense camera 67 × 67 mm, from Photonic Science) at two sample-to-detector distances (112 and 68 mm) with an exposure time of 80 seconds. Data were gathered from samples with aligned monodomain texture to benefit from the induced director alignment and have the distinct scattering patterns. The scattering patterns were processed and analysed using ImageJ and OriginPro to determine the spacing of nematic and smectic ordering in our LCEs using the Bragg equation, as well as the orientational order parameter.

Dynamic mechanical analysis (DMA) was performed using a TA instrument Discovery DMA850 in tensile mode. Film-shaped samples cut from a thin film with a cross-section of 0.8 × 2 mm were used for this characterization. Samples were cycled at 1 Hz with a strain amplitude of 0.01% and data were collected from −40 to 80 °C with a 3 °C interval and a soak time of 2 minutes at each temperature to ensure equilibration. We found this an important aspect of DMA measurement in the ‘temperature sweep’ mode, because the more common ‘T-ramp’ mode, which does not permit sample equilibration during data collection, does not show crystallization, which will be an important point of the discussion below.

## 3 Results and discussion

In this study, three mesogenic acrylate species were used: RM23, RM105 and CB3A, each having distinct molecular features expected to influence the liquid crystalline ordering in the elastomer. Two of the mesogens had a strong longitudinal dipole moment, arising from the –CN group, which often gives the propensity to form head-to-head dimers; one of the mesogens had a much shorter aliphatic tail, compared to the other two, with the odd number of carbons expected to impart an angle on the mesogen attachment;^29^ one of the mesogens had a more compact biphenyl mesogenic core, compared to the other two.

With the highly incompatible siloxane backbone, the propensity for micro-phase separation is strong, which results in a lamellar (smectic) structure: layers of mesogenic rods aligned perpendicular to the layer plane, with the backbone and spacers compacted into thin sheets between the layers. The relatively long flexible C–C chain between the backbone and the mesogenic rods also facilitates the formation of smectic layers. The mesogens were specifically chosen as two pairs out of the three candidates share similarities in their chemical structure, thus giving us an opportunity to study the compatibility between mesogens when mixed together. The mixing of mesogens also serves as a way of disrupting the smectic layering and offering a possibility for the nematic side-chain LCE material, in contrast with a uniform smectic layer.

### 3.1 Phase transformation

**Fig. 4:**
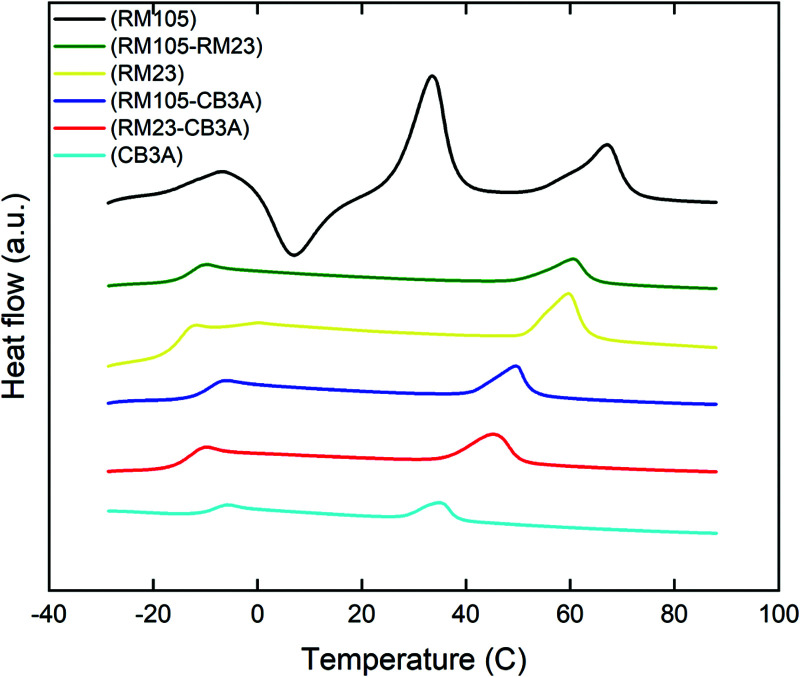
DSC heating curves of the side chain LCE samples. The curves (in all cases collected from the second heating cycle) are coloured based on the mesogen species in each sample: light blue for CB3A; black for RM105; yellow for RM23; green for RM105–RM23; dark blue for RM105–CB3A; and red for RM23–CB3A.

The phase transformation in our LCE materials is characterised by differential scanning calorimetry (DSC) and the results are shown in Fig. 4 with the transition temperatures listed in Table 1. All samples went through three heating–cooling cycles with excellent reproducibility of DSC curves, indicating high thermal stability. The DSC curves include two main features: a step corresponding to the glass transition temperature and a peak corresponding to the smectic–isotropic phase transformation temperature (the smectic phase was identified using XRD, see Section 3.3 below). However, the sample with pure RM105 also showed evidence of crystallization (also confirmed by DMA and X-ray analysis, later in the paper). The crystallization and melting transition peaks can also be seen on its heating curve above the *T*_g_. The immediate occurrence of crystallization after glass transition made it difficult to determine *T*_g_ itself, so another DSC scan with an annealed sample (thus with no crystallization and melting) was done to identify the glass transition temperature.

**Table 1:** List of the glass transition temperature *T*_g_ and the isotropic phase transition temperature *T*_c_ for all six mesogen compositions studied

Mesogen species	*T*_g_ (°C)	*T*_c_ (°C)
CB3A	−9	27
RM23	−15	50
RM105	−12	59
CB3A + RM23	−14	36
RM23 + RM105	−14	51
CB3A + RM105	−10	41

Due to the flexible Si–O links in the siloxane backbone, all samples have a much lower glass transition temperature when compared to other hydrocarbon elastomers. All *T*_g_ values were recorded between −15 and −9 °C. When mixing different mesogen species, both the glass transition temperature *T*_g_ and the smectic–isotropic phase transition temperature *T*_c_ shifted to the middle point of their parent mesogen species.

### 3.2 Linear dynamic response

**Fig. 5:**
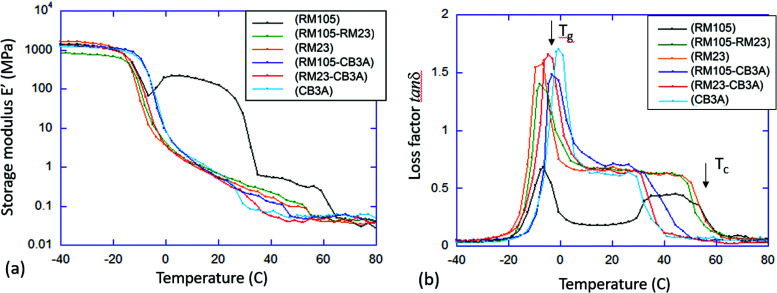
Dynamic mechanical analysis of xLCE. (a) The linear storage modulus *E*′ (a) and the loss factor tan*δ* (b) measured at a constant frequency of 1 Hz, are plotted against temperature. The curves are marked by the mesogen species in each sample: CB3A; RM105; RM23; RM105 + RM23; RM105 + CB3A; and RM23 + CB3A. The arrows indicate the glass and the isotropic transition points that have a very clear signature in the DMA signal.

To examine the thermal–mechanical properties, dynamic mechanical analysis (DMA) in tensile mode was carried out on all six materials at 1 Hz with a strain amplitude of 0.01%, which ensures the linear response. The temperature range was selected to be from −40 to 80 °C to sufficiently accommodate both the glass transition and the liquid crystalline phase transition. To obtain more accurate results, the “temperature sweep” method was adopted in which the sample was equilibrated at each target temperature (at 3 °C intervals) for 2 minutes before data acquisition. The storage modulus *E*′ (Young modulus) and the corresponding loss factor tan*δ* were plotted against temperature as shown in Fig. 5a and b, respectively.

Similar to DSC, DMA can also be used to identify the transition temperatures *T*_g_ and *T*_c_. *T*_g_ was determined by the first peak in tan*δ*, while the point of transition to isotropic phase, *T*_c_, was determined by the final characteristic drop in *E*′ as well as the return of the loss factor tan*δ* to low values characteristic of isotropic rubber. The results from DMA agree with the order of transition temperatures found in DSC, although DMA generally provided higher values especially for *T*_g_. This is due to the inherent difference between the two techniques, caused by the different position of temperature sensors and insulation; thus, these data only serve as a comparison to verify the trend of transition temperatures found in DSC.

As mentioned in Section 3.1, the elastomer synthesized with only RM105 can crystallize above *T*_g_ (with the subsequent melting) upon heating. This can be clearly seen in both Fig. 5a and b. Starting with an annealed sample, crystallization begins soon after exiting the glass phase at around −5 °C, resulting in the rise of the modulus *E*′ and the drop of tan*δ*. As temperature increases, melting occurs, leading to the drop of *E*′ and the rise of tan*δ*. On observing both graphs, it is obvious that both *E*′ and tan*δ* would exhibit a similar shape on heating in all other materials where crystallization does not occur.

### 3.3 Structure

The structure of our LCE materials was characterized by first performing X-ray analysis at room temperature. The aim of this experiment was to identify the type of orientational ordering and distinguish between nematic and smectic phases, and also to extract three important parameters: the orientational order parameter *Q*, the nematic order spacing *a* and the smectic order spacing *d*. The side-chain smectic A phase in the LCE network is constructed by many rod-like mesogenic units, each having one end attached to a siloxane chain. The long spacing between the rods and the polymer chain allows the formation of smectic layers in planes between the packed mesogens. In each layer, the mesogenic units sit parallel to each other, as well as the layer normal, resembling a tightly packed bookshelf. Therefore, *a* refers to the average lateral spacing between two adjacent mesogenic rods and *d* refers to the average spacing between the layer planes.

To detect this structure by X-ray analysis, all materials must be aligned before analysis. Traditionally, the alignment is achieved by applying an external stress, but it was proven to be a difficult proposition in polydomain smectic LCEs, because of many strong internal constraints imposed by layers and domain walls. However, we could reliably form an aligned monodomain texture by applying a uniaxial stress in the high-temperature isotropic phase, and then cooling into the smectic phase under stress, so that the anisotropic phase is formed effectively under a uniaxial (internal) field. This forces the nematic director to be aligned, and the smectic layers follow this uniform alignment by forming perpendicular to the imposed mechanical field. In this way, the monodomain smectic-A structure becomes ‘locked’, since the internal layer constraints would dominate over the weaker entropic forces of the network strands. This is confirmed by the clear transparent uniaxially-birefringent appearance of the samples ‘programmed’ in this way, in contrast to the usual opaque light-scattering appearance of polydomain samples. After forming such an aligned smectic-A LCE, we are able to characterize it by X-ray analysis, and also examine subsequent structural transformation that takes place on further mechanical stretching.

**Fig. 6:**
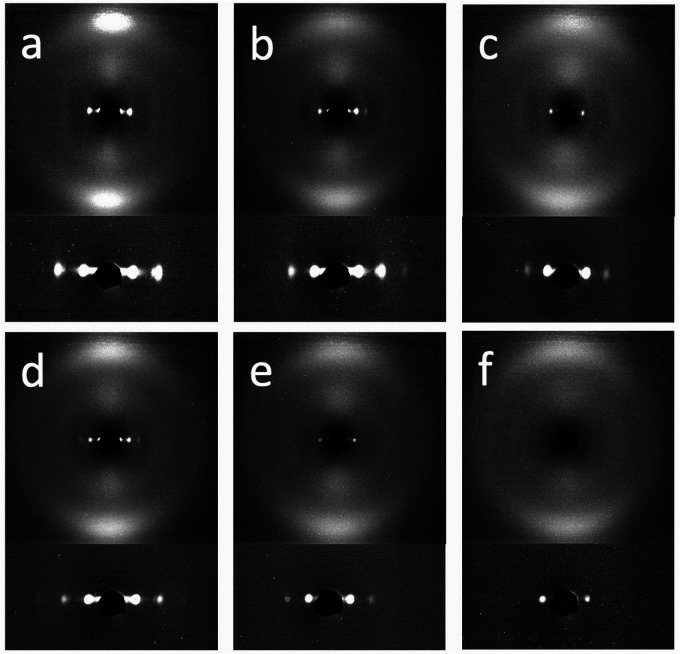
XRD patterns of the aligned samples synthesized from (a) RM105, (b) RM105 + RM23, (c) RM23, (d) RM105 + CB3A, (e) RM23 + CB3A and (f) CB3A. In each panel, the small-angle scattering pattern is magnified and shown below the wide angle pattern.

Two sample-to-detector distances were used to explore the wide-angle and small-angle scattering regimes. The longer sample-to-detector distance (small-angle scattering) helped to reveal more smectic layer signals that would otherwise be obscured by the beam stop. The XRD patterns of all six materials (all in the aligned state) are presented in Fig. 6, from which *a*, *d* and *Q* were calculated. These parameters are listed in Table 2. Note that we do not show the crystalline order in the RM105 material, which would form after a certain time after cooling from the isotropic phase (certainly, overnight).

**Table 2:** Values of the nematic order parameter *Q*, the nematic order spacing *a*, and the smectic order spacing *d*

Mesogen species	*Q*	*a* (Å)	*d* (Å)
CB3A	0.66	3.91	38.8
RM23	0.55	3.91	43.7
RM105	0.73	3.87	34.4
CB3A + RM23	0.61	3.90	42.6
RM23 + RM105	0.67	3.93	38.5
CB3A + RM105	0.69	3.91	36.4

Both the nematic and smectic order spacings are closely related to the chemical structures of the mesogenic units used in the synthesis, in particular, the ‘rod-like’ portion (mainly the aromatic section) in the mesogen molecule (measured by *b* in Fig. 2), and the flexible tails of the mesogen (*c*) and of the thiol moiety of the backbone (8 Å). Adding these dimensions for the non-polar mesogen RM105, we have the ‘expected’ smectic layer spacing of *d* = 33.8 Å, which is very close to what we find in X-ray analysis. In stark contrast, the smectic layer spacing is much longer in the highly polar CB3A and RM23 than their added molecular dimensions (talking about the pure materials first), see Table 2. This systematic discrepancy has to be related to the strong longitudinal dipole moment of the –CN group, which is known to form dimers in liquid crystal phases. Considering the mixtures, we also see the same systematic trend, where the CB3A + RM23 is the material where all mesogens carry a strong dipole moment, and accordingly a much large *d* spacing. The increase in the layer spacing in the other two mixtures (involving the non-polar RM105) is noticeable, but less pronounced, compared with the single-layer spacing of pure RM105. Note that the mesogen thickness (or more accurately, the lateral spacing *a*) is almost the same for all mesogens, which corresponds to the same angular position of the wide-angle reflection in Fig. 6.

**Fig. 7:**
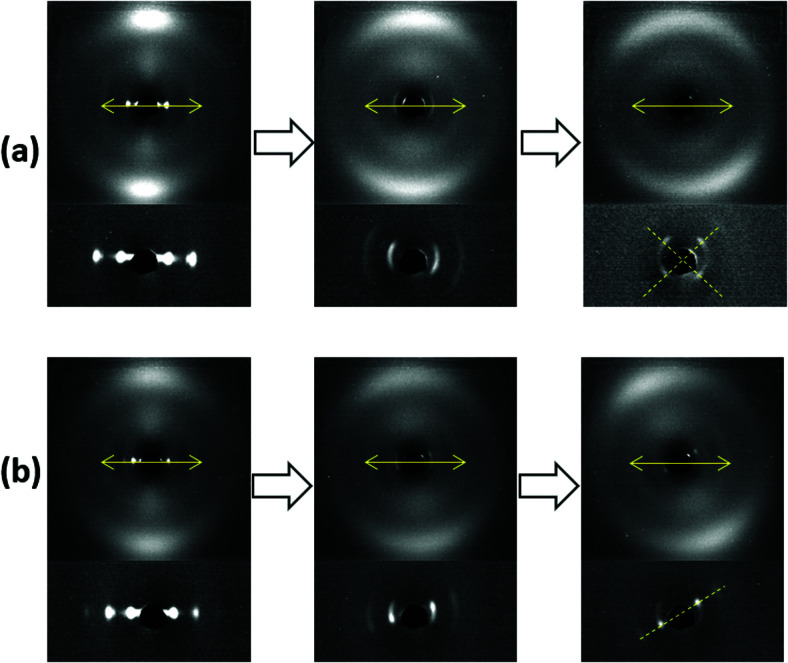
XRD patterns of aligned samples synthesized from (a) RM105 and (b) RM105 + RM23, with the increasing degree of stretching from left to right (starting with no stretching). The stretching direction along the nematic director and the layer normal is marked on all wide-angle XRD patterns by double-headed arrows. In the last stage of stretching, the diagonals on which smectic-layer signals reside are marked by dashed lines.

It is also interesting to examine the XRD patterns when the aligned samples are stretched parallel to their layer normals (Fig. 7). Both samples showed a similar XRD pattern initially, in which the nematic outer ring and the orthogonal smectic signals became diffuse with a slight rotation in the nematic director and the smectic layer normal. Upon further stretching, very profound discrepancies were observed. The sample with RM105 alone showed an even more diffuse nematic ring as well as four diagonal smectic signals. In contrast, the sample with RM105 + RM23 showed more concentrated signals in general and both the nematic director and the layer normal have rotated significantly.

**Fig. 8:**
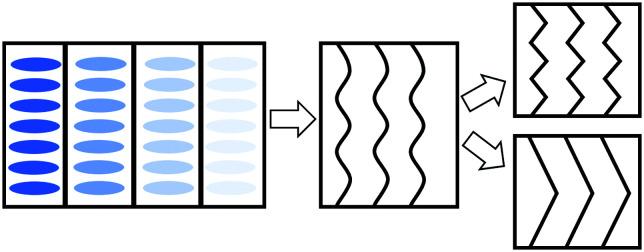
A scheme of the deformation mechanisms upon further stretching the aligned samples parallel to their smectic layer normals. The blue ellipses represent the mesogen “rods”, but we focus on layer planes in the subsequent images. The black lines inside each box represent the smectic layers. Left: The standard side chain LCE network structure. Middle: wave-like Helfrich–Hurault instability upon stretching. Right: Eventually, the smectic layers can either form micro zig-zags (top), or form much larger stripe domains.

In order to explain the observations above, a schematic drawing was made to better visualize the two distinct deformation mechanisms (Fig. 8). Initially, stretching along the layer normal causes the classical Helfrich–Hurault instability, generating a microscopic wave-like pattern.^16,30^ This corresponds to the broadened nematic and smectic reflections in the XRD patterns in the first stage of stretching. When stretched even further, the layer deformation is coarsened, and we find two distinct patterns and underlying deformation mechanisms. For one, the microscopic wave-like pattern keeps developing, eventually giving rise to a “zig-zag” pattern where the layer normal is separated into two distinct directions.^25,26^ As for the other mechanism, the microscopic Helfrich–Hurault pattern merges into macroscopic domains with a single consistent angle of layer (and the director) rotation. We see this as a distinct pattern rotation in the XRD image, Fig. 7b. However, when we move the X-ray beam spot (of about 1 mm diameter) to a different position across the sample, we find this rotation in the opposite direction, so we conclude that there are two kinds of ‘stripe domains’ with layer/director rotation, and the associated local shear. This is the ‘stripe domain’ mechanism caused by the incompatibility of local shear and the flat sample clamps, known in nematic LCEs.^31^ Clearly, the sample with RM105 alone followed the first mechanism: the microscopic zig-zags resulted in the four diagonally placed smectic spots, with each pair corresponding to one of the two layer normals, as in the Nishikawa and Finkelmann studies.^26^ Because of this frequent layer normal alteration, the nematic alignment was heavily disrupted, resulting in a significantly broadened outer ring. On the other hand, the sample synthesized with RM105 + RM23 eventually formed the macroscopic stripe domain pattern, where the domain sizes exceed the X-ray beam spot; thus, only one pair of rotated smectic signals were observed and both nematic and smectic signals appeared more concentrated. These two are the most representative of the XRD patterns evolving on deformation; other samples frequently had a combination of both effects. We associate this distinction with the strength of the smectic order (which we only assess indirectly *via* its correlation to the underlying nematic order) and the associated layer-network confinement,^15^ with the RM105 material having the strongest coupling to the layers – while other materials have this order lower.

## 4 Conclusions

The well-established thiol–acrylate ‘click’ reaction of Michael addition was used to synthesize liquid crystalline elastomers with the side-chain configuration. Three mesogenic acrylate species, namely RM105, RM23 and CB3A were examined, both on their own and in 50 : 50 mixtures, all prepared at a fixed 15% crosslinking density. This gave us an opportunity to study the effect of the mesogen chemical structure on the phase transformation behavior, thermal–mechanical properties and internal phase structure of the resulting LCEs. Given the difficulty in aligning side chain LCEs by mechanical stress, the samples were aligned by cooling from isotropic while stretched, forcing the nematic directors to be aligned, and the smectic layers forming flat planes perpendicular to it.

The structural study of LC ordering by XRD confirmed the smectic-A phase in all cases (which is expected, given the long spacers and the incompatible siloxane backbone), with both *a* and *d* spacings closely related to the physical size of the mesogen molecules. The lateral mesogen packing *a* in all cases is almost the same, corresponding to the lateral size obtained from the chemical structure. The layer spacing *d* for non-polar mesogens (RM105) is the smallest, and correlates to the mesogen dimensions. However, for polar mesogens involving the strong longitudinal dipole of the –CN group, we found that the layer spacing much increased, suggesting the dimer head-to-head formation. In the mixed polar–nonpolar systems, the increase in *d* spacing was intermediate.

We further found a difference in structural changes on uniaxial stretching along the layer normal (the initial director alignment axis). Depending on the strength of the smectic order, we found either a microscopic zig-zag Helfrich–Hurault pattern or a macroscopic stripe domain rotation of layers. This also correlates to the values of the mechanical loss factor, which was unusually high in the smectic phase for materials with weaker layer confinement (almost reaching the ‘nematic’ level of tan *δ*).

The combination of fast and robust ‘click’ chemistry and off-the-shelf ingredients makes thiol–acrylate side-chain LCEs widely available for even non-specialists and opens up new doors for side-chain LCE research. In terms of potential applications, the smectic-A LCEs are generally not considered as best actuators; however, the strong shape-memory effect arising from the network-layer coupling and constraints is a potent feature of smectic LCEs and appears to be the most promising property of these materials. Also, the ability to program, and re-program, the alignment patterns by cooling from the isotropic state under stress (also due to strong layer-network constraints) is a potentially applicable feature.

## Conflicts of interest

There are no conflicts to declare.

## Supplementary Material
